# Effects of platelet-rich plasma combined with exercise therapy for one year on knee osteoarthritis: retrospective cohort study

**DOI:** 10.1186/s13018-024-05186-w

**Published:** 2024-10-28

**Authors:** Tsuneo Kawahara, Shuhei Iida, Kazuma Isoda, Sungdo Kim

**Affiliations:** 1 Mizue Orthopedic Clinic, 4 Chome-45-1 Mizue, Edogawa-ku, Tokyo, 132-0011 Japan; 2 Faculty of Health and Medical Sciences, Department of Physical Therapy, Teikyo Heisei University, 2 Chome-51-4 Higashi-Ikebukuro, Toshima-ku, Tokyo, 170-8445 Japan

**Keywords:** Platelet-rich plasma, Exercise therapy, Knee osteoarthritis

## Abstract

**Background:**

Platelet-rich plasma (PRP) is a promising treatment for knee osteoarthritis (OA). However, exercise therapy and activities of daily living (ADL) guidance are recommended as core treatments in the Osteoarthritis Research Society International (OARSI) guidelines. However, the effects of PRP combined with exercise therapy are not fully understood. This study aimed to clarify the effectiveness of this treatment.

**Methods:**

We assigned patients diagnosed with knee OA and treated between January 2021 and December 2022 to groups who underwent PRP + exercise (PE), PRP (P), or exercise (E) therapy. Outcomes were evaluated using Knee Injury and Osteoarthritis Outcome Scores (KOOS) before, and 1, 3, and 12 months after treatment. Within-group comparisons according to the time of each score were statistically assessed using a one-way analysis of variance, then differences were analyzed using Bonferroni multiple comparisons *p* < 0.05). Treatment responses were determined using Outcome Measures in Rheumatology (OMERACT)-OARSI Responder criteria.

**Results:**

Pre-treatment KOOS did not significantly differ among the groups. Pain in the PE group improved within 1 month, symptoms, ADL, and quality of life (QOL) improved after 3, months and continued for 12 months. Pain and symptoms improved in the P group within 1 month, but ADLs and the QOL did not significantly change. Pain improved after 3 months in the E group and ADL, and QOL improved by 12 months. The response among the groups was the highest for the PE, with 50.0% at 1 and 3 months, and 65.0% at 12 months.

**Conclusions:**

Therapy with PRP immediately relieved pain, whereas exercise conferred late, but enduring effects. Combining PRP with exercise conferred synergistic advantages that persisted for up to 12 months.

## Background

Platelet-rich plasma (PRP) exerts anti-inflammatory properties by modulating the canonical nuclear factor κB signaling pathway in various cell types, including synoviocytes, macrophages, and chondrocytes [1, 2]. The cellular and molecular mechanisms underlying this potential therapeutic effect are not yet fully understood [1, 2]; however, they have gradually emerged in recent years [3].

Initially, PRP was used for bone graft regeneration in oral surgery, and it is now applied in areas such as cosmetics, sports, and orthopedics [4–6].

The ability of PRP to promote healing in soft tissues such as the rotator cuff, Achilles tendon, ligaments, and bones has been established in orthopedic surgery [7–9]. Although evidence about the healing properties of PRP and certain knee diseases is contradictory [10], PRP does exert short-term therapeutic effects on knee osteoarthritis (OA) [1, 11–14]. Consensus at the European Society of Sports Traumatology, Knee Surgery and Arthroscopy (ESSKA) in 2022 reported that PRP is effective [15]. Furthermore, the effectiveness of PRP depends on the severity of knee OA [16]. Treatment is usually applied for up to one year, and follow-up has continued for 5 years [17].

Exercise for patients with knee OA was considered a core treatment in the 2014 and 2019 Osteoarthritis Research Society International (OARSI) guidelines [18, 19]. Patient education was classified as a core treatment, although randomized controlled trial (RCT) data were lacking. The most recent guidelines for medical practice in Japan consider patient education as useful [20].

There are several existing reports on the combined treatment effects of PRP and exercise therapy [21, 22]. However, both studies were limited to a short duration of just 6 months.

The therapeutic effects of PRP have been assessed for up to one year, but the long-term effects of the combination with exercise after one year require investigation. The effectiveness of PRP combined with exercise one year after treatment remains unclear. Therefore, this study aimed to determine the effects of PRP combined with exercise for up to 1 year after treatment.

## Methods

We treated patients diagnosed with knee OA between January 2021 and December 2022. Patients with inflammatory arthritis, acute trauma, previous lower limb fracture or surgery, and the inability to continue treatment were excluded. A doctor presented these treatments to the patients during regular consultations, and the patients decided which option to choose. We then assigned 56 patients into groups that were treated with PRP and exercise (PE; *n* = 20), PRP (P: *n* = 16), or exercise (E; *n* = 20).

**Fig. 1:**
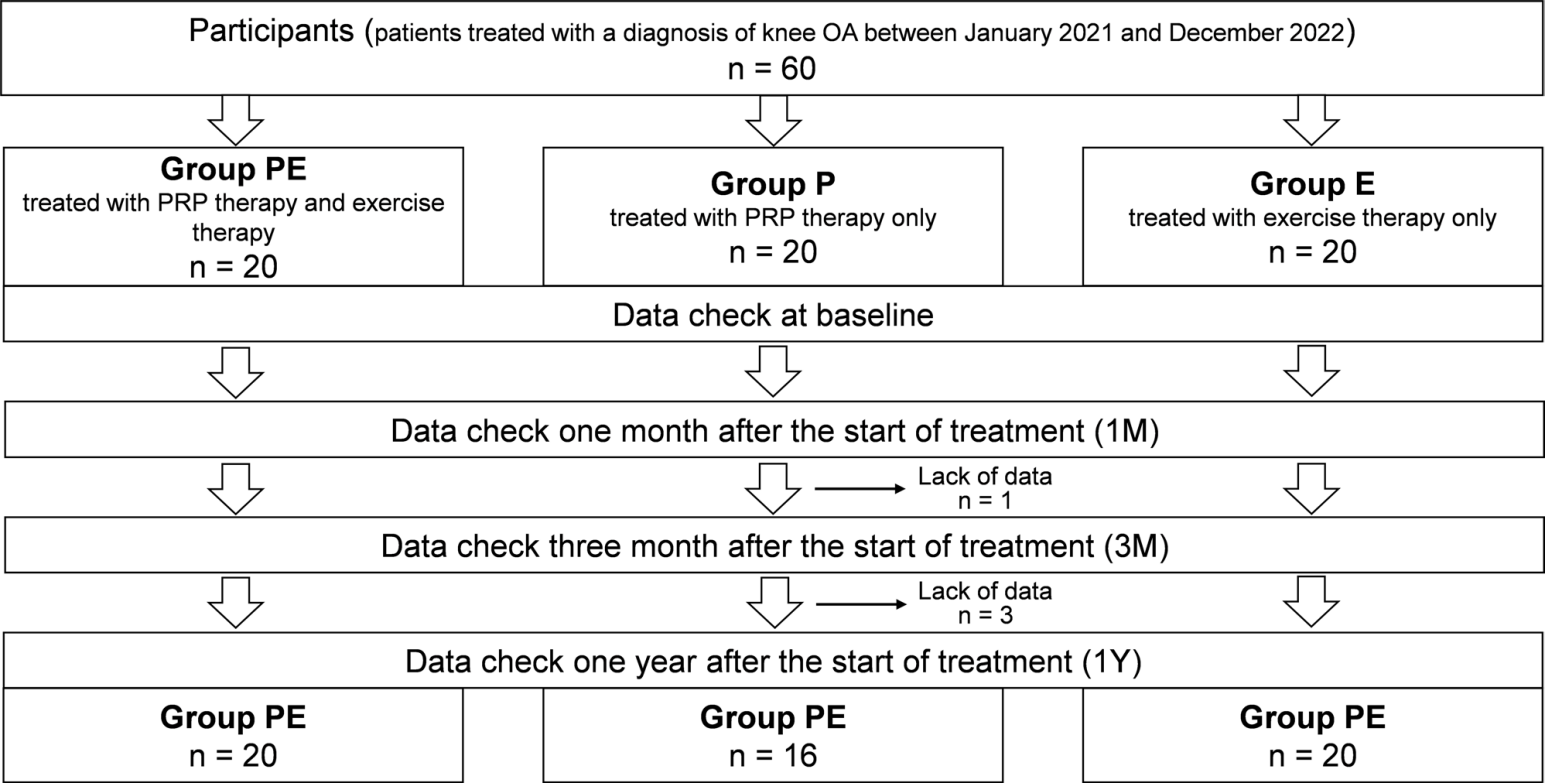
Flowchart of patients assigned to each group and data collection. Patients were divided into three treatment groups, and data were collected at baseline, 1 month, 3 months, and 1 year after treatment

Intentional influence from the doctor was strictly avoided. The patients were allocated to groups as described [22], and Fig. 1 shows a flowchart of the study.

We used MyCells^®^ Platelet Rich Plasma harvesting kits (https://my-cells.net/) and centrifugation at 3,500 rpm for 7 min to generate leukocyte-poor PRP from blood samples provided by the patients.

We interviewed the patients to specifically determine the types of movement they routinely used, then implemented appropriate physiotherapy to improve physical functions such as joint range of motion, muscle strength, and balance. The use of each body part and the location of the center of gravity (COG) were specifically modified from kinematic and kinetic perspectives, while patients implemented these movements once or two times per week for an average of five months.

Evaluations included Knee Injury and Osteoarthritis Outcome Scores (KOOS) that consist of the following subscales: pain, symptoms, activities of daily living (ADL), sports, QOL, and test-retest reproducibility. We used the validated WOMAC that assesses the knee OA index in its complete and original form and correlates with the Medical Outcomes Study (MOS) short form 36 health survey (SF-36) and the Lysholm knee scale [23, 24].

The patients were surveyed before and at 1, 3, and 12 months after intervention.

**Fig. 2:**
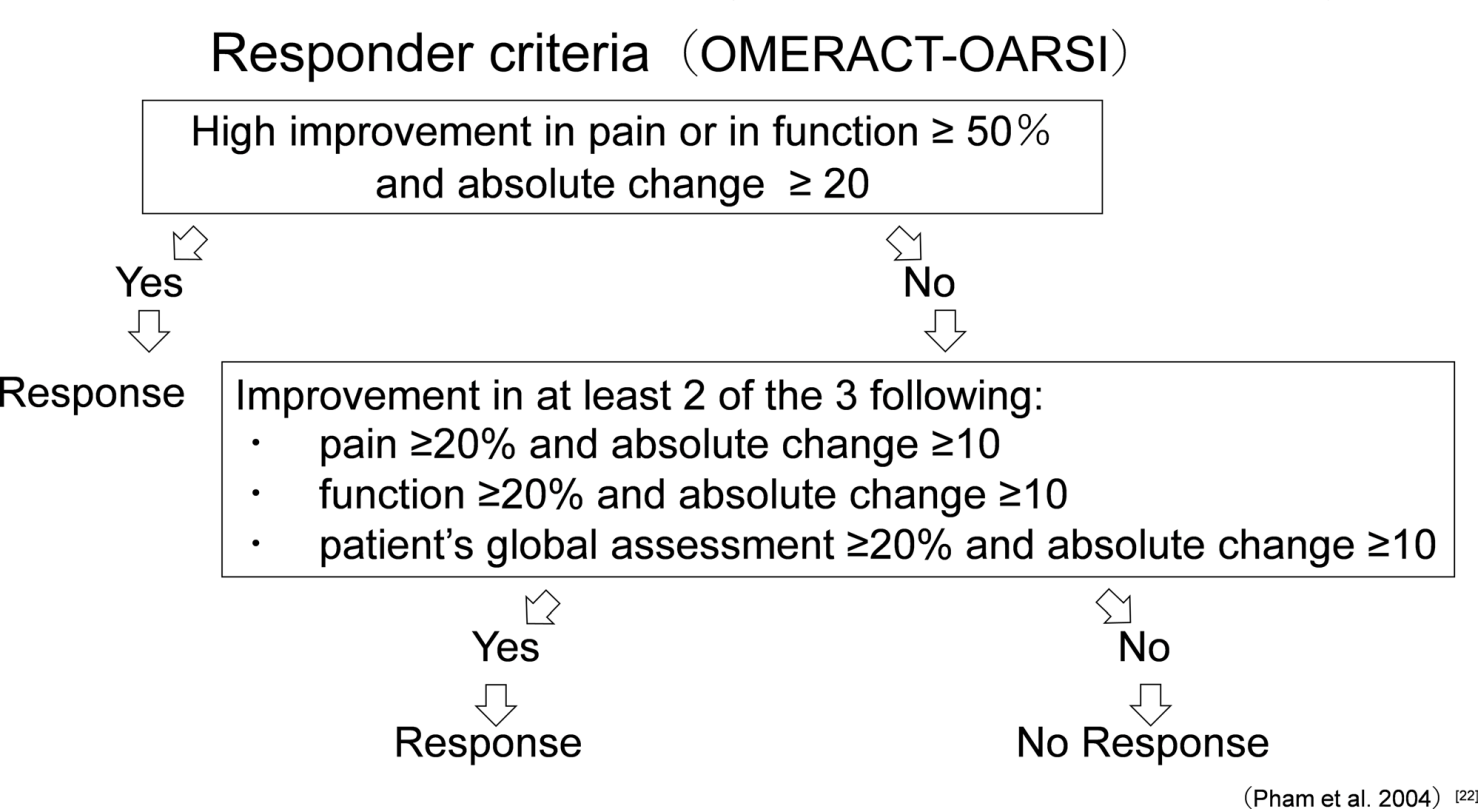
OMERACT-OARSI set of responder criteria. Flowchart of indicators defining patients who respond to treatment and improve. Modified and adapted from reference [22]

We assessed treatment outcomes using the Outcome Measures in Rheumatology (OMERACT) responder criteria from the OARSI, which were applied by Pham et al. to verify efficacy [25]. Figure 2 shows a flowchart of effectiveness assessments.

Data were statistically analyzed using Free JSTAT v. 13.0 (Vector Inc., Tokyo, Japan). Changes over time in each group and the amount of such changes between groups were assessed using a one-way analysis of variance. Significant differences were analyzed using Bonferroni multiple comparison tests (*p* < 0.05).

The Teikyo Heisei University Ethics Review Committee approved the study (Approval ID: 2024-022), which complied with the ethical principles enshrined in the Declaration of Helsinki (2013 amendment). All 56 patients who met the inclusion criteria provided written informed consent to participate in the study.

## Results

Data were collected for up to one year from 20, 16, and 20 patients in the PE, P, and E groups. One and three patients in the P group dropped out at 3 and 12 months, respectively, both due to insufficient data. Table 1 shows that the baseline data and KOOS values did not significantly differ among the groups.

**Table 1:** Basic and KOOS data at baseline in three groups

	PE	P	E
n	20	16	20
Age	69.5 ± 8.5	70.8 ± 10.9	74.7 ± 6.4
KL	II:11, III:7, IV;2	I:1, II:8, III:7	II:8, III:8, IV;4
Flexion ROM	124.2 ± 11.7	121.9 ± 10.9	127.0 ± 11.2
Extension ROM	-5.6 ± 5.1	-6.3 ± 5.0	-5.3 ± 5.3
KOOS baseline			
Pain	56.5 ± 20.0	62.5 ± 12.5	56.9 ± 12.2
Symptom	59.5 ± 18.0	67.6 ± 16.5	62.5 ± 16.2
ADL	74.0 ± 16.5	80.9 ± 12.0	71.0 ± 13.7
Sports	46.5 ± 25.8	55.3 ± 19.3	37.8 ± 17.4
QOL	42.8 ± 20.8	48.4 ± 14.5	41.6 ± 17.0
Total	61.6 ± 16.4	68.6 ± 10.9	59.8 ± 11.7

Values are shown as means ± standard deviation. ADL, activity of daily living; E, exercise; KL, Kellgren Lawrence; KOOS, Knee Injury and Osteoarthritis Outcome Score; P, platelet-rich plasma; PE, platelet-rich plasma and exercise; QOL, quality of life; ROM, range of motion

**Fig. 3:**
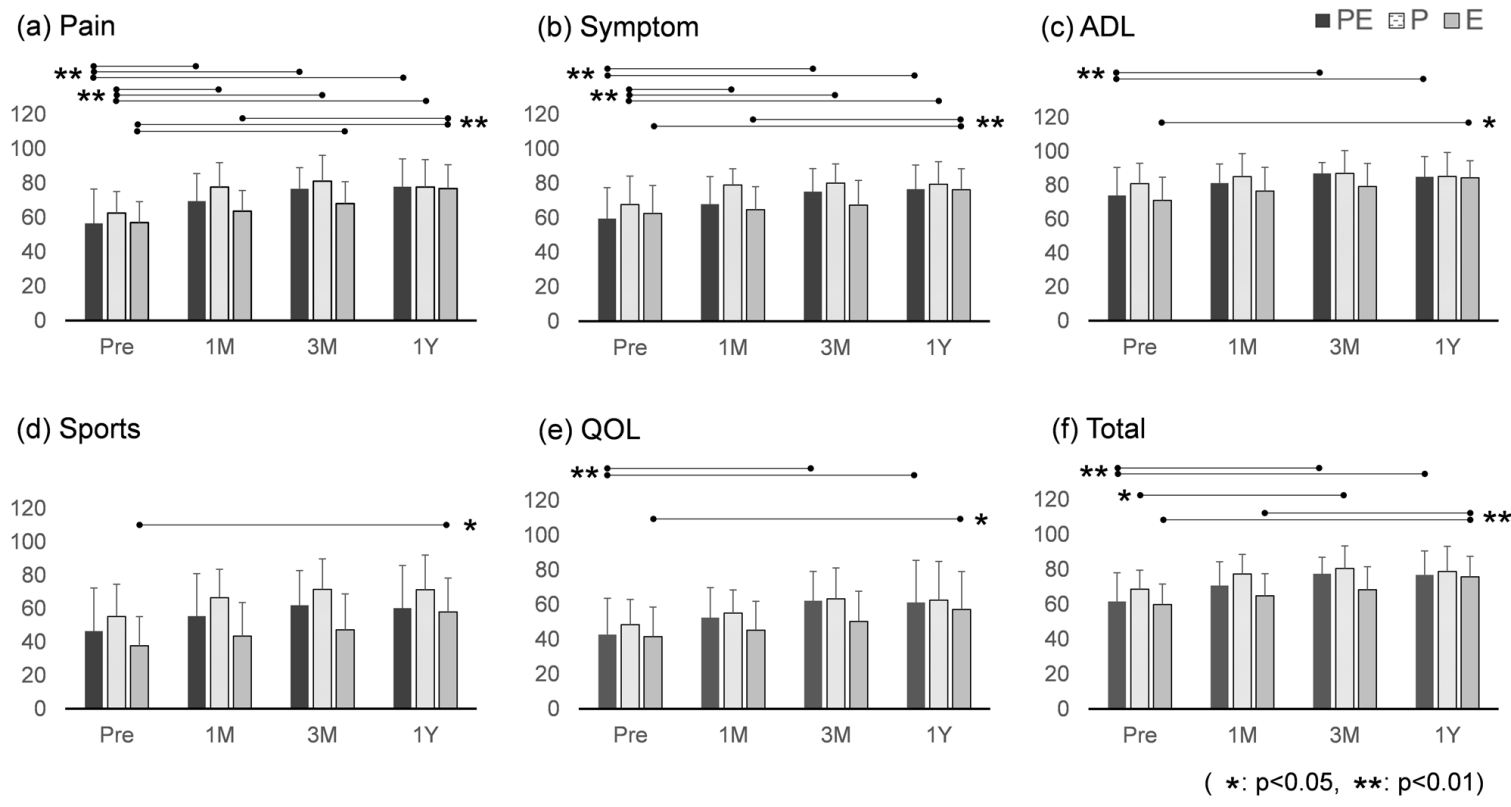
Changes in KOOS scores for each group. In each graph, the vertical axis shows the KOOS value, and the horizontal axis shows the time of each check. The black bars show the scores of the PE group, the dotted bars show the scores of the P group, and the grey bars show the scores of the E group. (**a**)–(**e**) Scores for each of the KOOS items: pain, symptoms, ADL, sports, and QOL, respectively; (**f**) shows the total score. The horizontal lines at the top of the graph connect statistically significant differences, with * indicating a p-value of less than 0.05 and ** indicating a p-value of less than 0.01. KOOS: Knee Injury and Osteoarthritis Outcome Score; ADL: Activity of Daily Living; QOL: Quality of Life

The scores for each of the KOOS items at each time period are shown graphically in Fig. 3. Pain improved after 1 month, and symptoms, ADL, and QOL improved after 3 months in the PE group and continued to improve for 1 year. Pain and symptoms improved after 1 month in the P group, but ADLs and QOL did not significantly change. Pain improved after 3 months, and all items improved after 1 year in group E (Table 2).

**Table 2:** KOOS for PE, *P*, and E groups at 1, 3, and 12 months

	PE	P	E
**Month 1**			
Pain	69.4 ± 16.0	77.6 ± 14.3	63.6 ± 12.1
Symptom	67.9 ± 16.0	79.0 ± 9.4	64.6 ± 13.4
ADL	81.3 ± 11.3	85.0 ± 13.6	76.5 ± 14.0
Sports	55.5 ± 25.4	66.6 ± 17.0	43.5 ± 20.1
QOL	52.5 ± 17.3	55.1 ± 13.4	45.3 ± 16.6
Total	70.7 ± 13.7	77.4 ± 11.2	64.9 ± 12.7
**Month 3**			
Pain	76.7 ± 12.3	81.1 ± 15.0	68.1 ± 12.7
Symptom	75.2 ± 13.3	80.1 ± 11.1	67.3 ± 14.3
ADL	86.9 ± 6.5	86.9 ± 13.5	79.3 ± 13.6
Sports	62.0 ± 20.8	71.6 ± 18.2	47.3 ± 21.6
QOL	62.2 ± 16.9	63.3 ± 17.8	50.3 ± 17.4
Total	77.4 ± 9.5	80.5 ± 12.9	68.3 ± 13.1
**Month 12**			
Pain	77.9 ± 16.1	77.6 ± 16.0	76.8 ± 13.8
Symptom	76.6 ± 14.0	79.5 ± 13.0	76.3 ± 12.2
ADLs	84.9 ± 12.0	85.2 ± 14.1	84.4 ± 10.0
Sports	60.3 ± 25.6	71.3 ± 20.9	58.0 ± 20.3
QOL	61.3 ± 24.2	62.5 ± 22.4	57.2 ± 21.9
Total	76.8 ± 13.7	78.8 ± 14.4	75.7 ± 11.8

Values are shown as means ± standard deviation. ADLs, activities of daily living; E, exercise; KOOS, Knee Injury and Osteoarthritis Outcome Score; P, platelet-rich plasma; PE, platelet-rich plasma and exercise; QOL, quality of life

**Fig. 4:**
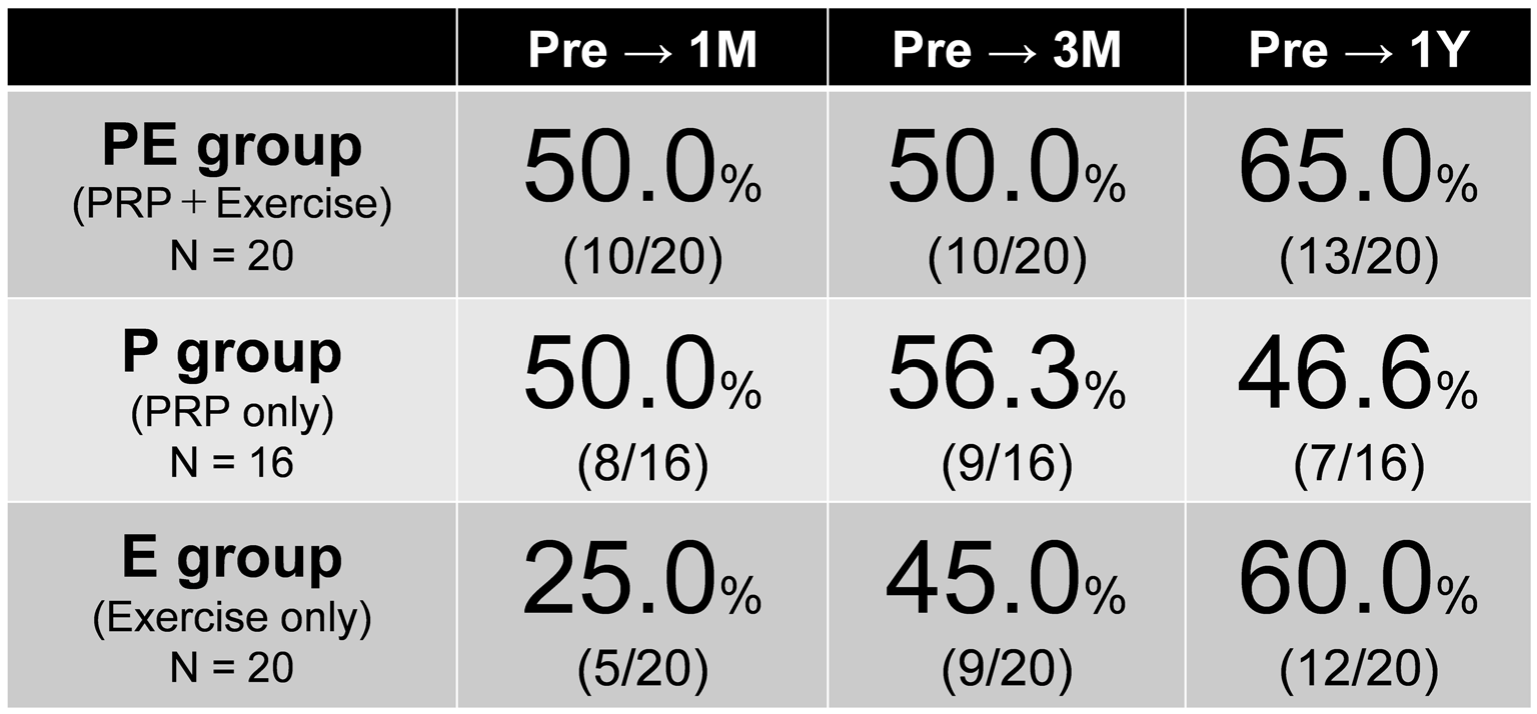
Results of OMERACT-OARSI Responder Criteria. Percentage of responders based on OMERACT-OARSI responder criteria in each group. OMERACT: Outcome Measures in Rheumatology; Osteoarthritis Research Society International

Figure 4 shows treatment responses based on responder criteria. At 1 month, 50.0%, 50%, and 25% of the PE, P, and E groups positively responded to treatment.

At 3 months, 50.0%, 56.3%, and 45.0% in the PE, P, and E groups positively responded to treatment. The response rate did not increase in the PE group but improved in the other groups.

At 1 year, the responses of 65.0% and 60.0% of the PE and E groups improved, whereas those of the P group declined to 46.6%. The improvement came later for the E group.

## Discussion

We assessed the effects of PRP combined with exercise for up to 1 year after intervention.

That exercise is effective against knee OA has been established. The latest OARSI guidelines (2019) offer comprehensive and patient-centered treatment profiles for persons with knee, hip, and polyarticular OA, which facilitates individualized treatment decisions for OA management [19].

These guidelines describe structured land-based exercise programs, dietary weight management combined with exercise, and mind-body exercises (such as Tai Chi and Yoga) to be effective and safe for all patients with knee OA, regardless of comorbidities [19].

Aquatic exercise is supported by a modest evidence base and confers robust benefits on pain and objective measures of function, and it was previously recommended alongside land-based exercise in the guidelines. However, aquatic exercise received a conditional recommendation due to accessibility issues, financial burden, and uptake concerns [19], and numbers of specific therapeutic exercise sets were not specified.

This omission might have been associated with the need to tailor exercise regimens to the individual physical characteristics and social circumstances of patients. We believe that patient education is important in this respect. In clinical practice, patients proceed only after providing a written, informed consent form. Thereafter, they were given monthly written instructions outlining knee OA that detail changes in their symptoms and prescribed physiotherapy regimens including a self-training program.

Patient education is considered a standard treatment according to the OARSI guidelines, despite the lack of RCT data [19]. Considering the individuality of patients and the influences of various living environments, reference can be made to the Japanese practice guidelines.

The most recent edition of these guidelines (2023) [20] affirms the effectiveness of exercise therapy and patient education in managing knee OA. Patient education includes instructions for real-life and work-related activities. Familiar and routine daily movements and weight distribution might inadvertently exacerbate symptoms. Detecting abnormal movement is important according to the Sahrmann kinesiopathological model of the movement system [26]. Motor skill training (MST) is effective in terms of teaching movement in real-life scenarios [27]. Although that study [27] focused on patients with low back pain (LBP), those with chronic LBP who underwent MST had greater short- and long-term functional improvements compared with those who practiced strength and flexibility exercises (SFE). Therefore, person-specific MST should be tailored to the functional activities limited by chronic LBP [27].

The current findings indicated that the benefits of exercise therapy are delayed. This might be attributed to the fact that exercise therapy at the clinic was provided for 40 min once or two times weekly. Therefore, motor learning required over 1 month to even begin to improve proprioception and movement, with the effects becoming evident after 3 months and continuing for 1 year.

We observed that PRP therapy for knee OA delivered anti-inflammatory and pain-relieving effects after 1 month. These results were comparable to previous findings. According to the most recent consensus by ESSKA, PRP is effective for patients with mild to moderate OA (KL grade ≤ 3), as indicated by Grade A evidence. Injected PRP provides better and longer-lasting symptomatic improvement compared with hyaluronic acid injections (Grade B) and steroids. Moreover, PRP therapy is not chondrotoxic (Grade A) and regenerates cartilage. Disease-modifying effects in experimental animals have been investigated only once, and evidence regarding humans is scant (Grade C) [15]. Therapy with PRP is primarily positioned as a treatment for symptomatic improvement in knee OA.

That PRP relieves pain concurred with previous findings, but the long-term effects of PCP slightly decreased at 1 year of treatment. This is a limitation of PRP therapy, and future studies are needed to determine the most effective platelet concentration and optimal frequency of treatment. A few of our patients received several PRP treatments. We therefore used the timing of the first PRP treatment as the baseline and analyzed the data as a single group. The KOOS values for the P group were slightly higher at baseline. However, the difference was not statistically significant, suggesting that the treatment effect was less apparent than in the other groups.

Furthermore, PRP combined with exercise therapy seemed to be the most efficient, which aligned with previous findings. The powerful anti-inflammatory and pain-relieving properties of PRP are thought to promote ease of engaging in active exercise therapy at the early stages of treatment. Badr et al. reported that PRP combined with exercise therapy outperformed PRP or exercise therapy alone, indicating an additive effect. However, their data were limited to 6 months after treatment [22], and the authors emphasized the need for more investigation to optimize the prescription and use of PRP combined with t-exercise therapy.

This study has several limitations. The outcomes were limited to a simple physical function assessment of a small sample and patient-oriented assessment (KOOS), which did not provide more detailed insights into treatment effects, such as the assessment of cartilage quality using MRI. We cannot rule out the possibility that the missing samples contained important factors. We analyzed retrospective data that could not take the form of a randomized controlled trial (lack of randomization, blinding, and a priori protocols). The follow-up period was limited to 1 year. Knee OA is chronic, and 1 year after treatment falls within the short- to medium-term category. Longer-term follow-up results are necessary for a better perspective.

## Conclusion

Platelet-rich plasma immediately affected pain, whereas the effects of exercise therapy became obvious later but lasted longer. The combination of PRP with exercise therapy offered synergistic advantages and the potential to be most effective for up to 1 year after treatment.

## Data Availability

No datasets were generated or analysed during the current study.

## Abbreviations

ADLs: activities of daily living
ESSKA: European Society of Sports Traumatology, Knee Surgery & Arthroscopy
KL: Kellgren Lawrence
KOOS: Knee Injury and Osteoarthritis Outcome Score
LBP: Low Back Pain
MRI: magnetic resonance imaging
MST: motor skill training
OA: osteoarthritis
OARSI: Osteoarthritis Research Society International
OMERACT: Outcome Measures in Rheumatology
PRP: platelet-rich plasma
QOL: quality of life
RCT: randomized controlled trial
SFE: strength and flexibility exercise
WOMAC: Western Ontario and McMaster University osteoarthritis
